# Immunogenicity and reactogenicity of the adjuvanted respiratory syncytial virus vaccine in patients with chronic kidney disease

**DOI:** 10.1093/ckj/sfag093

**Published:** 2026-03-24

**Authors:** Richard Radun, Saskia Bronder, Amina Abu-Omar, Danilo Fliser, Martina Sester, David Schmit

**Affiliations:** Department of Internal Medicine IV, Saarland University Medical Center, Campus Homburg, Homburg, Germany; Department of Transplant and Infection Immunology, PharmaScienceHub (PSH), Saarland University, Homburg, Germany; Department of Internal Medicine IV, Saarland University Medical Center, Campus Homburg, Homburg, Germany; Department of Internal Medicine IV, Saarland University Medical Center, Campus Homburg, Homburg, Germany; Department of Transplant and Infection Immunology, PharmaScienceHub (PSH), Saarland University, Homburg, Germany; Center for Gender-specific Biology and Medicine (CGBM), Saarland University, Homburg, Germany; Department of Internal Medicine IV, Saarland University Medical Center, Campus Homburg, Homburg, Germany

**Keywords:** chronic kidney disease, immunoglobulins, respiratory syncytial virus, RSVpreF3-vaccination, T cells

## Abstract

**Background:**

Chronic kidney disease (CKD) contributes to global mortality and morbidity, also due to infectious complications resulting from immune system dysregulation. Recently, respiratory syncytial virus (RSV) vaccines based on the prefusion F (preF) glycoprotein have been licensed for prevention of severe disease in elderly and in patients with comorbidities, but data on immunogenicity in patients with CKD are scarce.

**Methods:**

We characterized humoral and cellular immunogenicity of 75 patients with CKD stages G3a to G5d both before and 14 days (IQR 2) after vaccination with an adjuvanted protein-based RSVpreF3-vaccine using ELISA and flow cytometry. Data on adverse events were collected through a self-reported questionnaire.

**Results:**

Vaccination led to a significant induction of RSV-specific CD4 T cells (*P* < .0001) and the increase did not differ between the CKD stages. CD8 T cells were not specifically induced. Despite high seroprevalence prior to vaccination, quantitative levels of RSV-specific immunoglobulins IgG and F protein-specific IgG were significantly induced on vaccination (both *P* < .0001), with a less pronounced increase in patients with advanced CKD. Urinary albumin-creatinine-ratio (UACR) was shown to be predictive of vaccine response in a multivariate regression model using age, serum creatinine and urea as covariates (*P* = .035). The vaccine was well tolerated with mostly transient adverse events at the injection site.

**Conclusions:**

RSV-vaccination led to a robust CD4 T-cell and humoral response in patients with CKD with less pronounced effects in those with high-grade proteinuria. Long-term data on immunogenicity and correlation with clinical outcomes are warranted to define optimal vaccination strategies.

## GRAPHICAL ABSTRACT

**GRAPHICAL ABSTRACT:**
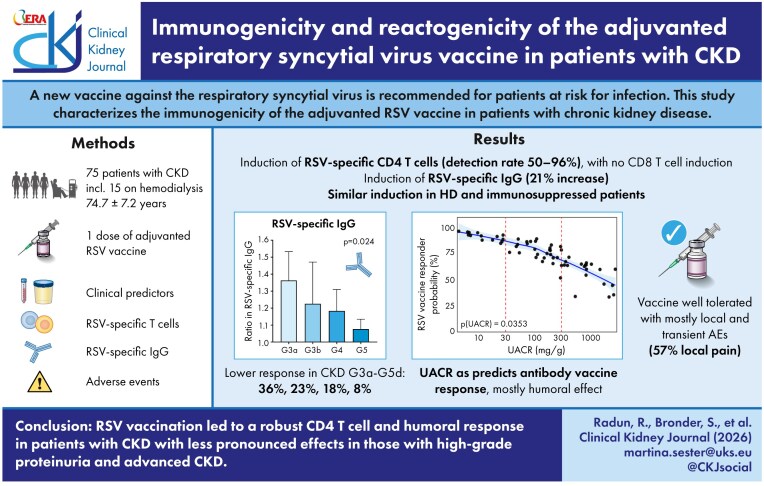

KEY LEARNING POINTS **What was known:**

- There are limited data on RSV-vaccine immunogenicity in patients with CKD, despite them being a high-risk group for developing severe RSV-associated lower respiratory tract infection.

**This study adds:**

- Administration of the AS01_E_-adjuvanted RSVpreF3-vaccine significantly induces RSV-specific IgG and CD4 T cells in patients with CKD, including those on hemodialysis and under medical immunosuppression.
- Humoral vaccine response is reduced in advanced CKD and inversely correlates with UACR.

**Potential impact:**

- RSV-vaccination is safe and immunogenic in patients with CKD, supporting the integration of RSV-vaccination into routine preventive care for the CKD population.

## INTRODUCTION

Respiratory syncytial virus (RSV) infection poses a major risk for developing virus-associated lower respiratory tract infections. In particular, high-risk groups including elderly and chronically ill individuals such as patients with chronic kidney disease (CKD) show high rates of RSV-associated complications with a global annual estimate of >5 million hospitalizations and 100 000 deaths worldwide [1]. Recently, RSV-vaccination strategies using protein-based or mRNA-based vaccine platforms have been licensed for elderly and individuals with comorbidities including patients with CKD [2]. Two international, randomized, placebo-controlled phase III trials using an AS01_E_-adjuvanted RSVpreF3-vaccine and the bivalent non-adjuvanted RSVpreF-vaccine have shown high vaccine efficacy of 94.1% and 85.7%, respectively, in preventing severe RSV-associated lower respiratory tract infection over the course of one RSV season in patients >60 years of age without relevant safety concerns [3, 4].

CKD affects ∼10% of the global population [5] and entails various facets of immunodeficiency. Innate and adaptive immunity are known to be impaired in uremia, and hemodialysis treatment as well as medical maintenance immunosuppression may further alter immune cell phenotype, antigen presentation and cytokine response. Reduced vaccine responsiveness in patients with CKD has been demonstrated for influenza, hepatitis B, and pneumococcal vaccines [6–11], where patients with advanced CKD and those receiving dialysis mount weaker and shorter-lived immune responses compared to the general population [12]. Despite the recent surge in RSV-vaccine research, there are limited data on immunogenicity and reactogenicity of RSV-vaccination in patients with CKD.

We therefore explored the cellular and humoral immunogenicity of RSV-vaccination in patients with CKD KDIGO stages G3–G5. A subset of patients received maintenance immunosuppression or intermittent hemodialysis treatment. To this end, we characterized the early vaccine-induced RSV-specific T cells and immunoglobulins in 75 patients with various CKD etiologies before and after receiving the AS01_E_-adjuvanted RSVpreF3-vaccine and assessed reactogenicity using a self-reported standardized questionnaire in this prospective cohort study.

## MATERIALS AND METHODS

### Study design and subjects

From January 2025 to May 2025, patients with CKD (KDIGO stages G3a to G5d) were prospectively enrolled at the Saarland University Medical Center in Homburg, Germany. All patients received a single dose of the protein-based AS01_E_-adjuvanted vaccine RSVPreF3 (“Arexvy,” GSK) intramuscularly according to national vaccination recommendations. Further details on collected demographic and clinical data and routine laboratory parameters are given in the Supplementary Methods. The study was performed in adherence with the declaration of Helsinki and approved by the ethics committee of the Saarland Medical Association (reference 99/24), and written informed consent was obtained from all individuals.

### Quantification and characterization of RSV-specific CD4 and CD8 T cells

RSV-specific CD4 and CD8 T cells were quantified and characterized using flow cytometry after a 6-hour stimulation of heparinized whole-blood samples as previously described [13, 14]. A detailed description of the procedure can be found in the Supplementary Methods including antibodies used for staining (Table S1), and a gating strategy for flow-cytometric analyses (Fig. S1).

### Determination of RSV-specific antibodies

Specific IgG and IgA antibodies toward pan-RSV and RSV-F (fusion protein) specific IgG were measured as described in the Supplementary Methods.

### Statistical analysis

All statistical analyses were performed using GraphPad Prism v.10.6 software (GraphPad, San Diego. CA, USA). Statistical comparisons were conducted using nonparametric tests in case of nonnormality of data. The Mann–Whitney *U*-test was applied for comparisons between two independent groups, while comparisons of more than two unpaired groups were performed using the Kruskal–Wallis *H* test followed by Dunn’s *post hoc* test in case of reaching significance. For paired nonparametric data, the Wilcoxon signed-rank test was employed. Linear correlations between immunological and clinical data were assessed using Spearman’s rank correlation coefficient, and multivariate logistic regression analysis was performed using R v.4.5.1 software to determine predictors of immunogenicity. Owing to the lack of predefined clinically relevant cutoffs, a composite vaccine response encompassing both RSV-specific CD4 T cells and IgG antibodies was defined in this study as any measurable rise from baseline. Parametric data were analyzed with an unpaired *t*-test, whereas nominal and dichotomous variables were compared using the chi-squared test. A two-sided significance level of *P* < .05 was considered statistically significant.

## RESULTS

### Study population

In this observational study, 75 patients with CKD were recruited with 17 to 21 patients in each CKD stage from G3a to G5d. Fifteen out of 19 patients within the CKD G5 cohort were on intermittent maintenance hemodialysis. Relevant demographic and clinical features including kidney disease, comorbidities, immunosuppression, laboratory parameters, and differential blood counts are shown in Table 1. The mean age of all patients was 74.7 years with no significant differences between groups and sex distribution was comparable across all groups. The median interval between vaccination and follow-up visit was 14 (IQR 2) days and did not differ between groups. There were no significant differences in the automated differential blood counts with regards to total leukocyte number among the patients except for a non-significant trend toward a higher number of monocytes in patients with CKD G5/G5d. Serum levels of creatinine, urea, and intact parathyroid hormone and UACR increased progressively with more advanced CKD. Conversely, hemoglobin levels and estimated glomerular filtration rate (eGFR) decreased. Eleven patients received B-cell or plasma cell-depleting therapy within the past 6 months (e.g. rituximab), ongoing treatment with calcineurin inhibitors, antimetabolites, glucocorticoids, or complement inhibitors. The prevalence of diabetes mellitus was similar in all groups whereas an autoimmune disease affecting the kidneys was more frequent in the group with advanced CKD. These included vasculitis, IgA nephropathy, membranous, or immune complex glomerulonephritis as well as renal amyloidosis.

**Table 1:** Clinical and demographic data on CKD study patients.

	Overall	G3a	G3b	G4	G5/G5d
	*n* = 75	*n* = 18	*n* = 21	*n* = 17	*n* = 19 (HD = 15)
Years of age, mean (SD)	74.7 (7.2)	72.1 (6.0)	73.7 (6.7)	76.7 (8.5)	76.3 (7.0)
Days between vaccination and follow-up measurement, median (IQR)	14 (2)	15 (4.5)	14 (1.5)	14 (1)	14 (5)
Sex, *n* (%)					
Female	32 (42.7)	9 (50)	7 (33.3)	6 (35.3)	9 (47.4)
Male	43 (57.3)	9 (50)	14 (66.7)	11(64.7)	10 (52.6)
Differential blood count, median (IQR) cells/µl					
Leukocytes	7600 (2600)	7550 (2500)	7200 (2500)	7500 (2200)	8200 (2600)
Granulocytes	5034 (2018)	4858 (1596)	4874 (1966)	5120 (2065)	5141 (2388)
Monocytes	676 (276)	720 (425)	640 (302)	646 (245)	770 (280)
Lymphocytes	1689 (730)	1866 (973)	1685 (504)	1550 (622)	1581 (676)
Hemoglobin, median (IQR), g/dl	12.9 (2.7)	13.5 (2.1)	14.1 (2.2)	12.8 (1.4)	11.4 (1.2)
Diabetes mellitus, *n* (%)	29 (38.7)	6 (33.3)	11 (52.4)	8 (47.1)	4 (21.1)
Renal autoimmune disease, *n* (%)	26 (34.7)	6 (33.3)	3 (14.3)	11 (64.7)	6 (31.6)
Immunosuppressive medication, *n* (%)	11 (14.7)	2 (11.1)	2 (9.5)	4 (23.5)	3 (15.8)
Serum urea, median (IQR) mg/dl	74 (55.5)	45.5 (20)	58 (26)	107 (24)	115 (55.5)
Serum creatinine, median (IQR) mg/dl	1.94 (1.63)	1.22 (0.15)	1.59 (0.31)	2.31 (0.75)	5.42 (3.28)
Creatinine-derived eGFR, median (IQR) ml/min/1.73 m²	30.8 (26.9)	51.7 (13.7)	36.6 (8.5)	24.3 (7.7)	7.1 (5.4)
Urine albumin–creatinine ratio, median (IQR) mg/g	142.2 (379.5)	26.3 (257.2)	52.7 (340.6)	137.1 (180.6)	289.2 (744.4)
Serum parathyroid hormone, median (IQR) pg/ml	88 (72)	47.5 (23.5)	74 (46)	93 (43)	161 (195)

Abbreviations: HD, hemodialysis; IQR, interquartile range; SD, standard deviation.

### Vaccine-induced humoral and cellular immunity in patients with CKD

**Figure 1:**
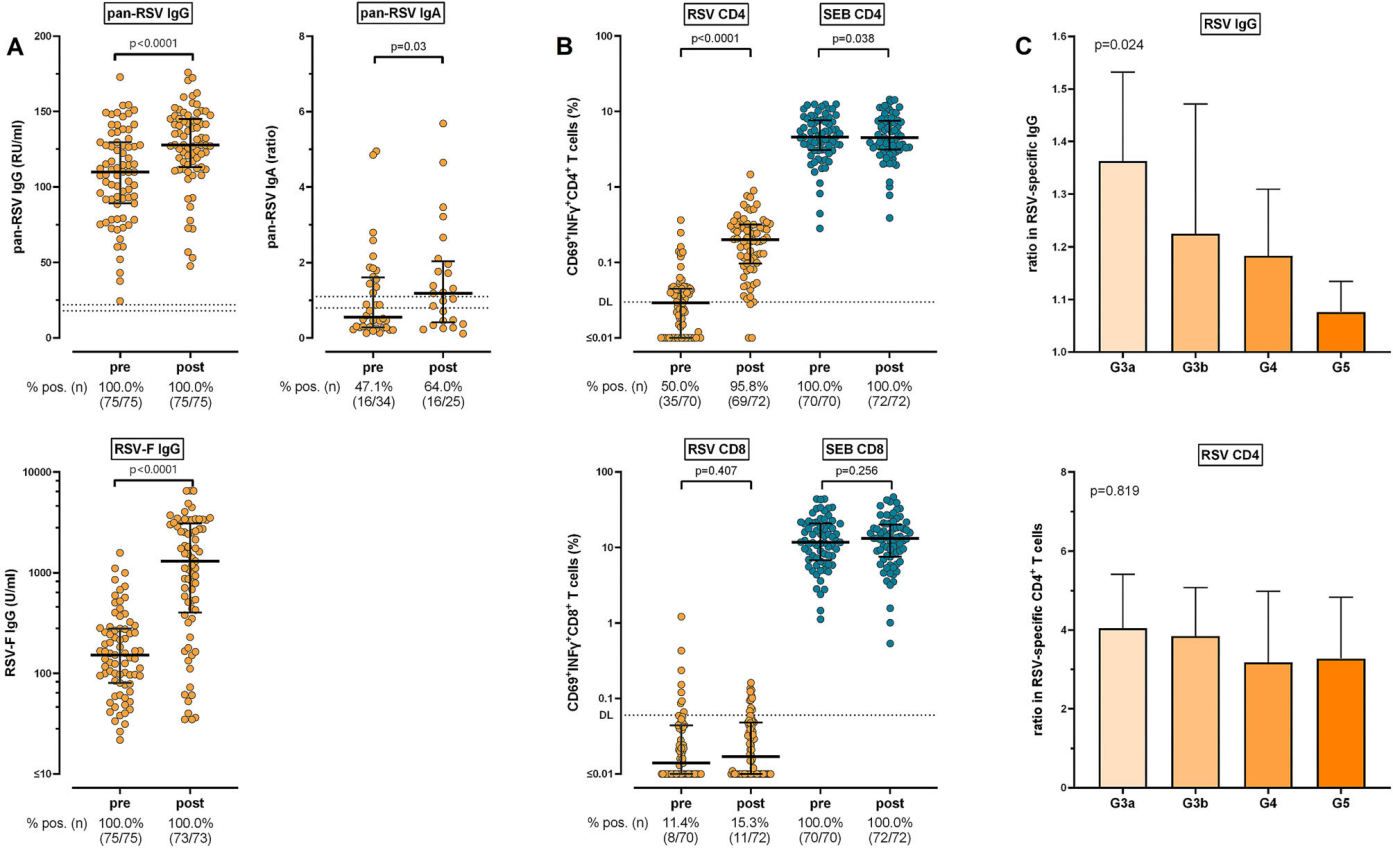
RSV-specific humoral and cellular vaccine response in patients with CKD. (a) Relative concentrations of RSV-specific IgG, IgA, and prefusion F-specific IgG before (pre-) and 14 days after (post-) vaccination with the adjuvanted RSVpreF3-vaccine (Arexvy), including corresponding percentages of seropositivity. The dashed line indicates the manufacturer’s cutoff for seropositivity (if available). Dots represent individual patients; lines represent medians and interquartile ranges. (b) Percentages of RSV-specific CD69^+^, IFNγ^+^ CD4, and CD8 T cells before (pre-) and 14 days after (post-) vaccination, including polyclonal stimulation with SEB and corresponding percentages of individuals above detection limits (DL, indicated by the dashed lines). Dots represent individual patients; lines represent medians and interquartile ranges. (c) Relative increase in cellular and humoral vaccine response for different CKD stages. Columns represent geometric means with 95% confidence intervals. *P* values were calculated from paired values using the Wilcoxon matched pairs test. (c) *P* values were calculated using the Kruskal–Wallis test. CD, cluster of differentiation; Ig, immunoglobulin.

For characterization of humoral immunogenicity of the RSVPreF3-AS01_E_-vaccine, RSV-specific IgG, IgA and F-IgG antibodies were measured. After a median follow-up of 14 days postvaccination, median pan-RSV-specific IgG levels showed a significant increase from 110 (IQR 41) RU/ml to 128 (IQR 32) RU/ml (*P* < .0001, geometric mean 1.21-fold Fig. 1a). Seropositivity defined as the percentage of individuals with an IgG level above 18 RU/ml was high with 100% before and after vaccination (Fig. 1a). Likewise, vaccine RSV-F protein-specific IgG antibodies were detectable already before vaccination, and showed a significant increase from 152 (IQR 198) U/ml to 1305 (IQR 2669) U/ml (5.99-fold, *P* < .0001). Pan-RSV-specific IgA levels were measured in a subgroup of patients. Despite low overall levels IgA levels showed a significant induction (1.51-fold, *P* = .030, Fig. 1a).

RSV-specific CD4 and CD8 T cells were quantified based on induction of interferon gamma (IFNγ) after stimulation with RSV-derived peptides. On vaccination, a significant increase in the percentage of RSV-specific activated CD4 T cells was observed (*P* < .0001, Fig. 1b). Before vaccination, 50% of patients showed RSV-specific CD4 T cells above the detection limit with an increase to 96% after vaccination (Fig. 1b). CD8 T cells showed higher variability at baseline and were not significantly induced upon vaccination (Fig. 1b). A pronounced CD4 and CD8 T-cell response was detected after polyclonal stimulation with *Staphylococcus aureus* enterotoxin B (SEB), which was largely unaffected by RSV-vaccination.

Differentiation of groups according to their clinical CKD stages revealed significant differences with highest geometric mean increases in pan-RSV-specific IgG titers in the G3a patient group with a 1.36-fold induction. In stages G3b, G4, and G5 the increases were 1.23-fold, 1.18-fold, and 1.08-fold, respectively (*P* = .024). Similarly, there was a numerical decline in RSV-specific CD4 T cells with higher CKD stages without reaching statistical significance (*P* = .819, Fig. 1c).

### Functional and phenotypical characterization of RSV-specific T cells

RSV-specific CD4 and CD8 T cells were further characterized for their capacity to produce the cytokines TNF and IL-2. As with T cells producing IFNγ, the percentage of CD69^+^ CD4 T cells producing TNF and IL-2 also showed a significant induction after vaccination, whereas respective CD8 T-cell populations were not induced (Fig. S2a). Among vaccine-induced RSV-specific CD4 T cells, most cells (54.9%) were polyfunctional with the capability to produce all three cytokines simultaneously, while 31.5% were dual-cytokine positive, and 13.6% of cells produced one cytokine only (Fig. S2b). This pattern was distinct from that of polyclonally stimulated T cells, where 19.5% of cells were triple positive and similar fractions produced two cytokines (43.5%) or one cytokine (37.0%). As a sign for recent antigen encounter, numerical expansion of RSV-specific CD4 T cells after vaccination was associated with a significant upregulation of CTLA-4 as compared to prevaccination levels (*P* < .0001). CTLA-4 expression on CD4 T cells after polyclonal stimulation was low and was unaffected by the vaccine (*P* = .304, Fig. S2c).

### Immunogenicity in CKD subgroups

**Figure 2:**
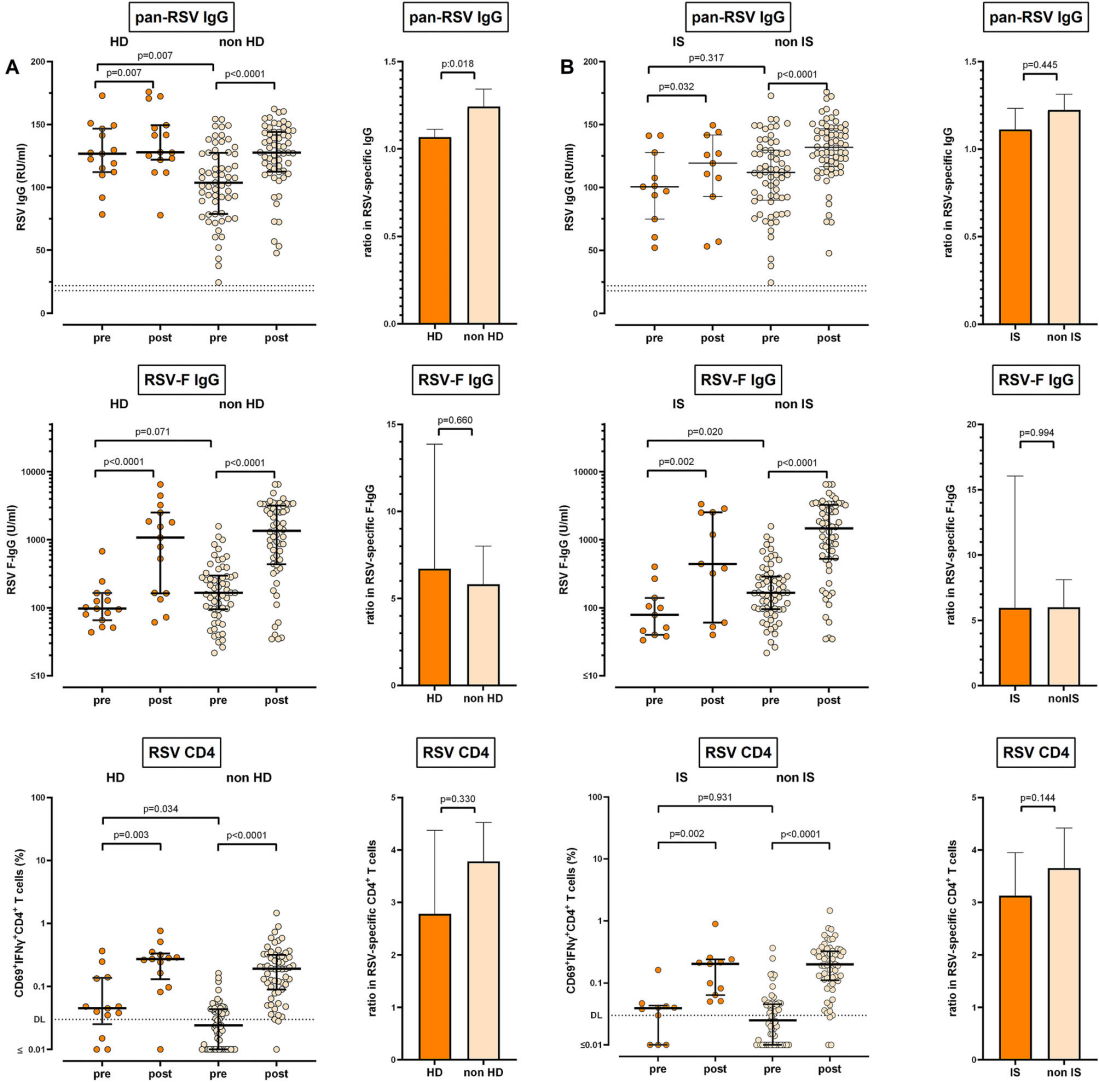
Differential cellular and humoral effects of RSV-vaccination in hemodialysis patients and immunosuppressed patients. Levels of pan-RSV-specific IgG and RSV-F-IgG, as well as percentages of RSV-specific CD4 T cells before (pre-) and 14 days after (post-) vaccination in (a) patients on intermittent hemodialysis (HD, *n* = 15) versus non-dialysis patients (non-HD, *n* = 60), and in (b) patients under medical maintenance immunosuppression (IS, *n* = 11) versus patients without medical immunosuppression (non IS, *n* = 64) including comparison of the fold increase on vaccination. Dots represent individual patients; lines represent medians and interquartile ranges; columns represent geometric means with 95% confidence intervals. *P* values were calculated from paired values using the Wilcoxon matched pairs test, *P* values for fold increases were calculated using the Mann–Whitney test. CD, cluster of differentiation; Ig, immunoglobulin.

We further assessed RSV-vaccine immunogenicity among CKD subgroups in exploratory analyses, where 15 patients on intermittent hemodialysis were compared with non-dialysis patients (Fig. 2a). Baseline levels of pan-RSV-specific IgG were higher in dialysis patients (*P* = .007). Levels increased significantly in both groups, but to a lesser extent in patients on hemodialysis than in non-dialysis patients (*P* < .018). By contrast, baseline levels of RSV-F-specific IgG were lower in patients on dialysis, and were robustly induced in both groups without significant differences in patients with and without dialysis (*P* = .469). Patients on dialysis had a higher proportion of RSV-specific CD4 T cells above detection limit at baseline (*P* = .034), which increased to a similar extent as in non-dialysis patients *P* = .330, Fig. 2a).

When comparing a subset of 11 patients with CKD receiving maintenance immunosuppressive therapy with patients without immunosuppression, both subgroups showed a significant increase in IgG antibodies (pan-RSV, F-specific) and RSV-specific CD4 T cells. However, there was no difference in the extent of increase between the groups (Fig. 2b). Finally, patients with CKD were stratified into an advanced disease cohort (eGFR <30 ml/min/1.73 m², >G4) and less advanced cohort (G3a/b). In both groups, RSV-specific IgG were robustly induced on vaccination with a less pronounced increase in advanced CKD (*P* = .030, Fig. S3). Similarly, a significant induction of RSV-F-specific IgG and RSV-specific CD4 T cells was observed in both groups, but the extent of increase did not differ between the groups (Fig. S3).

### Clinical correlations and predictors of immune response

**Figure 3:**
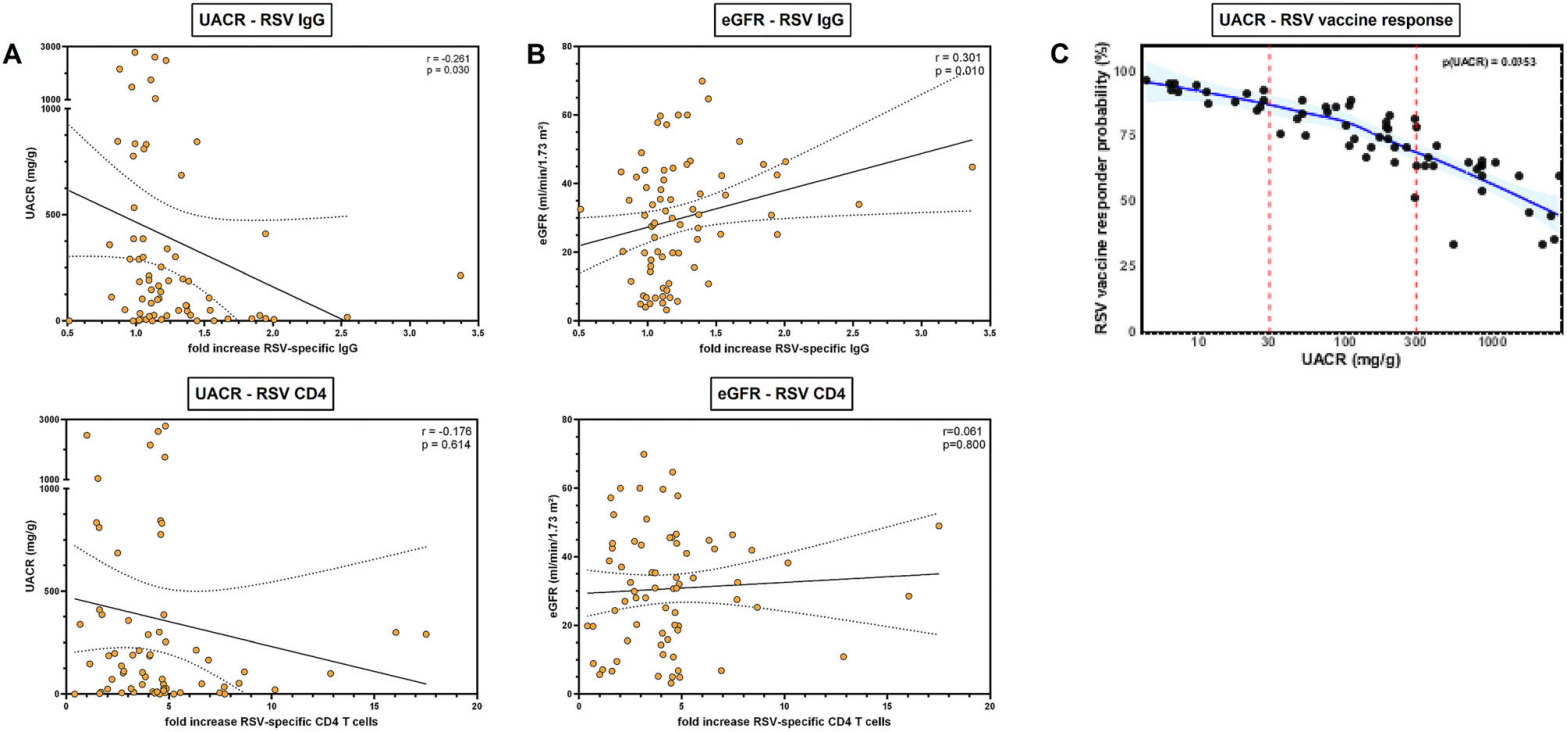
Correlations of humoral and cellular vaccine response with clinical CKD parameters prevaccination. (a) Linear inverse correlations between the fold increase in RSV-specific IgG or RSV-specific CD4 T cells and the UACR. Dots represent individual patients, orange-colored dots denote patients with immunosuppression. Lines correspond to the linear regression line with overlaid confidence intervals. (b) Linear correlation between fold increase in RSV-specific IgG and creatinine-derived eGFR. Dots represent individual patients. Lines correspond to the linear regression line with overlaid confidence intervals. (c) Predicted probability of being a vaccine responder based on a multivariate logistic regression model including log-transformed UACR as predictor and eGFR, serum urea and age as covariates. Due to the lack of predefined clinically relevant cutoffs, being a vaccine responder was defined as a composite of any measurable increase in RSV-specific CD4 T cells and IgG antibodies. The blue line represents a locally weighted scatterplot smoothing (LOESS) curve with other covariates set at the median, the light blue area displays the 95% confidence interval. Dots represent individual patients. CD, cluster of differentiation; Ig, immunoglobulin.

Standardized clinical parameters associated with CKD and its progression were collected and analyzed in terms of their correlation with cellular and humoral immunogenicity. A moderate but significant inverse linear correlation was found between the relative increase in RSV-specific IgG levels and the UACR at the time of vaccination (Spearman *r* = −0.261, *P* = .030, Fig. 3a). A similar inverse relation was found between the increase in RSV-specific CD4 T cells and UACR or C-reactive protein (CRP) as marker of systemic inflammation, but this did not reach statistical significance (UACR: Spearman *r* = −0.176, *P* = .614, Fig. 3a; CRP: *r* = −0.091, *P* = .441, Fig. S4a). eGFR correlated positively with RSV-IgG vaccine responses (Spearman *r* = 0.301, *P* = .03, Fig. 3b), in line with an inverse correlation of serum urea with RSV-IgG levels (Spearman *r* = −0.265, *P* = .024, Fig. S4a), respectively. In a multivariate logistic regression model a significant relationship between UACR and predicted vaccine responder probability defined as having an increase in both RSV-specific IgG and CD4 T cells was observed, showing significant decline of responder probability with high UACR levels (*P* = .035, Fig. 3c). By contrast, when CD4 T-cell or IgG responses were analyzed individually, no significant association with log-transformed UACR were observed despite a non-significant trend for humoral vaccine response (*P* = .412 for CD4 responses and *P* = .077 for IgG responses, Fig. S4b).

### Reactogenicity of RSV-vaccination in patients with CKD

**Figure 4:**
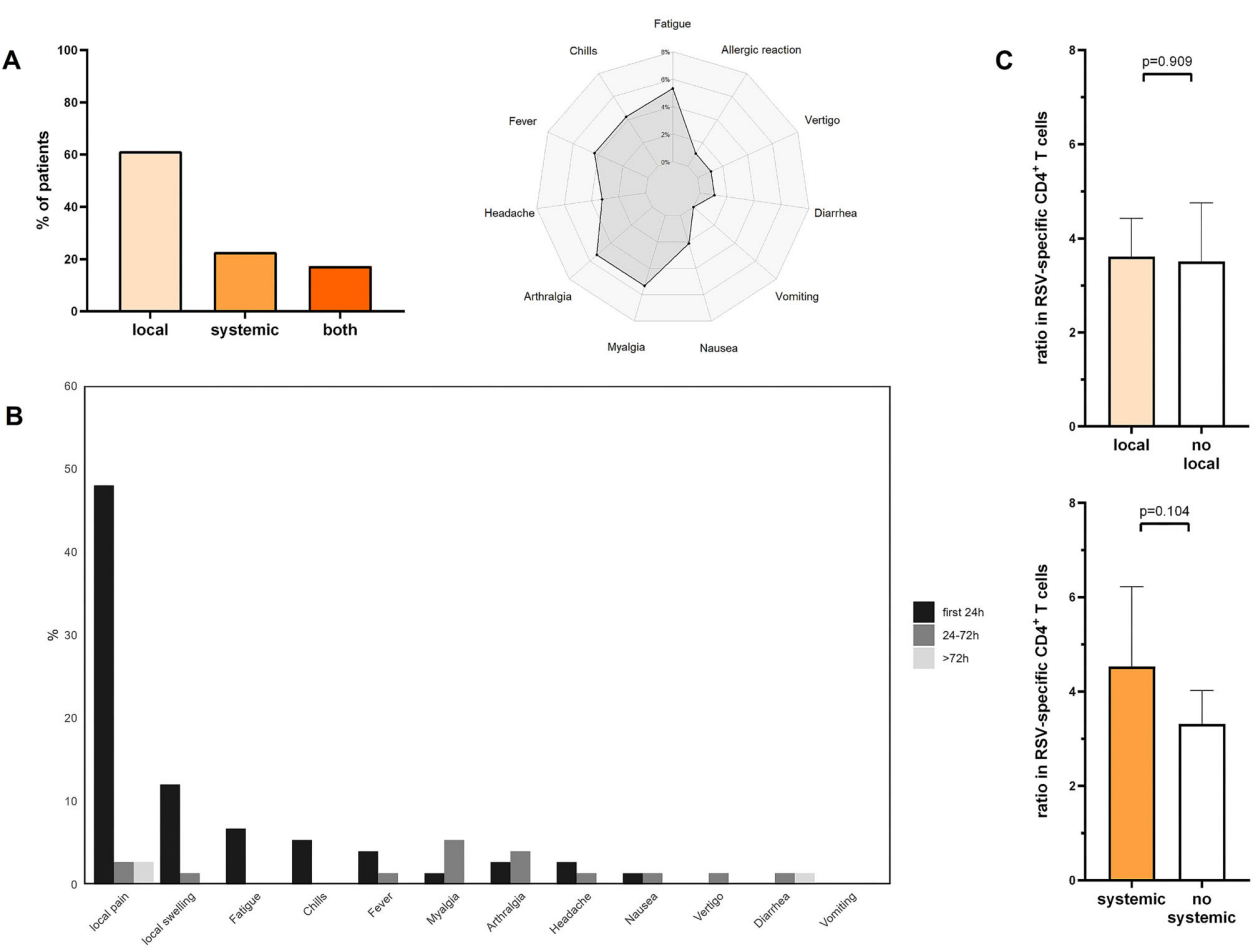
Reactogenicity of RSV-vaccination in patients with CKD. (a) Distribution of self-reported adverse events at different time points after vaccination. Bars represent percentage of patients. Radar plot illustrating systemic adverse events. Line represents percentage of patients with self-reported events . (b) Temporal distribution of self-reported adverse events within the first week after vaccination. Groups are stratified as first occurrence of symptoms within 24 hours, between 24 and 72 hours, or between 72 hours and 1 week after vaccination. Bars represent percentage of patients. (c) Fold increase in induction of RSV-specific CD4 T cells for patients with or without local or systemic reactions. Columns represent geometric means with 95% confidence intervals. Columns represent geometric means with 95% confidence intervals; *P* values were calculated using the Mann–Whitney test. CD, cluster of differentiation.

Reactogenicity was assessed in all patients using a standardized questionnaire on self-reported adverse events within the first 7 days. No adverse events were reported by 33% of patients, while 61% reported transient local reactions such as pain or swelling at the injection site. Twenty-three percent of patients stated systemic symptoms, most commonly arthralgia, myalgia, fatigue, or elevated body temperature (Fig. 4a and b). None of the symptoms persisted beyond 2 weeks or led to hospitalization. To evaluate a potential association between reactogenicity and cellular immune response, the relative increase in RSV-specific CD4 T cells was compared between patients with and without adverse events. However, no significant differences were observed (Fig. 4c).

## DISCUSSION

Due to a lack of specific antiviral therapies and risk for severe disease course in high-risk groups, RSV-vaccination poses a readily available preventive strategy for reducing RSV-associated morbidity and mortality. However, data on immunogenicity and reactogenicity of RSV-vaccination in patients with CKD are still scarce [2]. In line with global endemicity of RSV, we found a high rate of baseline seropositivity across all CKD stages. On vaccination with a single dose of the adjuvanted RSVpreF3-vaccine, a significant increase in pan-RSV-IgG and IgA, RSV-F-specific IgG as well as RSV-specific CD4 T cells was observed already after 14 days. There was no induction of RSV-specific CD8 T cells. The magnitude in the induction of humoral immune response was lower in patients on intermittent hemodialysis, while patients under maintenance medical immunosuppression showed similar cellular and humoral vaccine response as non-immunosuppressed individuals. An advanced CKD stage was associated with a lower humoral immunogenicity, and higher UACR was related to a lower vaccination response in a logistic regression model. RSV-vaccination was well tolerated and most patients reported only minor or transient local adverse events.

The study cohort had a mean age of 74.7 years and a median eGFR of 30 ml/min/1.73 m², representing a high-risk population for RSV-associated lower respiratory tract infections and comparable to other published CKD cohorts [15]. Patients were evenly distributed in terms of KDIGO stages, age, and sex, and their lymphocyte counts did not differ. Comorbidities were comparable across the groups with median prevalence of diabetes of 38.7%, kidney autoimmune disease of 34.7%, and immunosuppressive therapy of 15% [16].

In line with previous reports on RSV seroprevalence, RSV-specific immunoglobulins were detected in all patients at baseline [13, 17, 18]. On vaccination, pan-RSV-IgG, pan-RSV-IgA, and RSV-F-specific IgG significantly increased together with an increase in RSV-specific CD4 T cells as seen in other reports on immunocompromised individuals [13, 19, 20]. Similarly, recent reports found seroconversion in 61% of immunosuppressed patients [21]. Unlike for RSV-specific CD4 T cells, vaccination had no immediate effect on RSV-specific CD8 T cells. This aligns with previous findings on immunogenicity of adjuvanted RSVpreF3-vaccine and other protein-based vaccines such as the shingles-vaccine Shingrix or the COVID-19-vaccine NVX-CoV2373 [22–26]. This lack of effect can be attributed to the mechanism that peptides from protein-based vaccines are primarily presented through MHC class II therefore predominantly triggering a CD4 T-cell response [23].

For patients undergoing intermittent hemodialysis, induction of humoral immune response was significantly lower compared with non-dialysis patients in line with a numerically smaller increase in RSV-specific cellular immune response. Notably, this less pronounced increase in dialysis patients may be due to higher pan-RSV-specific IgG- and CD4 T-cell levels even before vaccination, which likely reflect prior exposure to RSV and underscores their increased infection risk. Despite exclusion of respiratory infection at the time of vaccination, subclinical RSV infection may have contributed to higher baseline levels. As the vaccine-induced immune response was studied early after vaccination and patients did not report any signs or symptoms of infection between vaccination and subsequent analysis, it is considered unlikely, that infections had a confounding effect on immunity measured after vaccination. Despite interindividual differences in the extent of vaccine-induced immune responses, pre-existing immunity may overall explain why a single dose is generally sufficient to induce a boost in immunity in all patients. In contrast, as inferred from COVID-19 vaccine studies [27, 28], one might speculate that a single dose of a vaccine would unlikely to be sufficient to induce a de novo induction of a primary RSV-specific immune response. Overall, our findings align with previous reports of impaired vaccine responses in dialysis patients, including lower antibody responses as well as faster waning of both cellular and humoral immune responses to vaccinations [6, 29, 30]. Similarly, patients under medical immunosuppression exhibited a numerically smaller increase in CD4 and RSV-specific IgG vaccine response without reaching statistical significance. This likely may result from the small sample size and heterogeneity of immunosuppressive regimens, including B-cell depleting antibodies and antimetabolites which are commonly known to impair humoral vaccine response [27, 31]. For the subgroup of immunosuppressed patients receiving complement inhibitors, effects of vaccine immunogenicity are poorly characterized. Preclinical studies suggest that C3b reduces complement activation and humoral response to adenovirus vectors [32], while clinical data indicate impaired antibody neutralization and altered T-cell memory development in viral infections such as influenza [33, 34]. RSV-vaccination immunogenicity has previously been studied in kidney and lung transplant recipients demonstrating strong cellular and humoral vaccine induction [13, 19, 20]. Future studies should address the stability of the vaccine immune response and effectiveness toward protection from RSV infection in both patients with CKD and solid organ transplant recipients.

Heterogeneity of cellular and humoral immune response prompted the question of defining predictive parameters of vaccine response. Interestingly, the increase in RSV-specific IgG but to a lesser extent of CD4 T cells showed an inverse correlation with UACR. Furthermore, there was a significant negative inverse correlation between RSV-specific IgG with serum urea levels as well as a significant positive correlation between RSV-specific IgG and eGFR in line with impaired immune function in uremia. In a multivariate logistic regression model including UACR, eGFR, serum urea, and age as covariates, an exploratory composite vaccine response probability defined as an increase of both RSV-specific IgG and CD4 T cells was in part predicted by prevaccination UACR. This effect was mostly attributable to the humoral vaccine response. There is limited data on vaccine immunogenicity in relation to proteinuria. In a small Japanese cohort, patients with nephrotic syndrome under medical immunosuppression mounted lower spike-specific antibody responses to SARS-CoV-2 vaccination [35]. In a small pediatric cohort with idiopathic nephrotic syndrome, reduced antibody titers against hepatitis B and tetanus were observed despite normal antigen-specific B-cell counts [36]. Although the reason for the diminished immune response in highly proteinuric patients is currently unclear, a possible explanation might be urinary loss of immunoglobulins or potentially even loss of vaccine antigen or adjuvants due to impaired filtration barrier. However, given intramuscular application and lymphatic transport to draining lymph nodes, systemic losses are likely of limited magnitude. Future studies are warranted to validate this observation and to evaluate potential underlying mechanisms responsible for an impaired vaccine response in highly proteinuric patients.

Vaccination using the adjuvanted RSVpreF3-vaccine was well tolerated with mostly transient and primarily local adverse events. Only a minority of patients reported systemic adverse events such as arthralgia or myalgia and no symptoms persisted beyond 7 days. This is in line with previous reports [3], including transplant recipients [13, 20]. Despite some associations between cellular immune responses and systemic adverse events found for other vaccines [37], no significant differences between patients with and without adverse events were found in terms of RSV-specific cellular immune response.

Study limitations are the single-center design without randomization, placebo control or blinding. The size of individual subgroups was relatively small with heterogeneity in comorbidities and medical immunosuppression, limiting the generalizability of our findings. Thus, the results of our subgroup analyses should be considered explorative. Furthermore, our study addresses early immunogenicity, and no data durability of RSV-specific immunoglobulins or on neutralizing activity were collected; together with observational data on effectiveness of the vaccine, this would be an important area for future research to increase clinical interpretability. As we did not systematically test for RSV infection, concurrent respiratory infection may have been present in some patients and contributed to interindividual variability of baseline- and/or vaccine-induced immunity. Larger studies are required to further delineate the role of pre-existing immunity for immunogenicity and durability of the vaccine response. Finally, findings from this study are based on *in vitro* stimulation assays and do not directly infer clinical vaccine effectiveness in terms of reduced infection risk or disease severity.

## CONCLUSION

A single intramuscular dose of the adjuvanted RSVpreF3-vaccine led to a significant cellular and humoral vaccine response in all patients with CKD. This held true for both patients under immunosuppressive therapy and intermittent hemodialysis treatment despite in part lower magnitude of effect in these subgroups. UACR was identified as one of the covariates for predicting vaccine response probability. The vaccine was well tolerated with predominantly local and transient adverse events. As our study focused on early immunogenicity, long-term stability of the immune responses and clinical data are warranted and careful consideration of reduced immunogenicity versus elevated infection risk and high infection-associated mortality remains essential to develop specific vaccination strategies in CKD cohorts.

## Supplementary Material

sfag093_Supplemental_File

## Data Availability

All figures and tables have associated raw data. The data that support the findings of this study are available from the corresponding authors upon request.
